# Clinical implications of intrinsic molecular subtypes of breast cancer for sentinel node status

**DOI:** 10.1038/s41598-021-81538-4

**Published:** 2021-01-26

**Authors:** Maria Rossing, Christina Bligaard Pedersen, Tove Tvedskov, Ilse Vejborg, Maj-Lis Talman, Lars Rønn Olsen, Niels Kroman, Finn Cilius Nielsen, Maj-Britt Jensen, Bent Ejlertsen

**Affiliations:** 1 Center for Genomic Medicine 4113, Copenhagen University Hospital, Blegdamsvej 9, 2100 Copenhagen, Denmark; 2 Department of Health Technology, Technical University of Denmark, Kongens Lyngby, Denmark; 3 Department of Breast Surgery, Rigshospitalet/Herlev-Gentofte Hospital, Copenhagen University Hospital, Copenhagen, Denmark; 4 Department of Radiology, Rigshospitalet, Copenhagen University Hospital, Copenhagen, Denmark; 5 Department of Pathology, Rigshospitalet, Copenhagen University Hospital, Rigshospitalet, Copenhagen, Denmark; 6 Danish Breast Cancer Cooperative Group, Rigshospitalet, Copenhagen University Hospital, Copenhagen, Denmark; 7 Department of Clinical Oncology, Rigshospitalet, Copenhagen University Hospital, Copenhagen, Denmark

**Keywords:** Cancer, Molecular medicine, Oncology

## Abstract

Axillary lymph node status is an important prognostic factor for breast cancer patients and sentinel lymph node biopsy (SLNB) is a less invasive surgical proxy. We examined if consecutively derived molecular subtypes from primary breast cancers provide additional predictive value for SLNB status. 1556 patients with a breast cancer > 10 mm underwent primary surgical procedure including SLNB and tumor specimens were assigned with a transcriptomics-based molecular subtype. 1020 patients had a negative sentinel node (SN) and 536 a positive. A significant association between tumor size and SN status (*p* < 0.0001) was found across all samples, but no association between size and SN status (*p* = 0.14) was found for BasL tumors. A BasL subtype was a predictor of an SN-negative status (*p* = 0.001, OR 0.58, 95% CI 0.38;0.90) and among the BasL, postmenopausal status was a predictor for SN-negative status (*p* = 0.01). Overall survival was significantly lower (*p* = 0.02) in patients with BasL tumors and a positive SN. Interestingly, we identified a significant correlation between hormone receptor activity and SN status within the BasL subtype. Taken together, molecular subtypes and hormone receptor activity of breast cancers add predictive value for SLNB status.

## Introduction

Axillary lymph node (ALN) status is a major prognostic factor in breast cancer and is accurately predicted by sentinel lymph node biopsy (SLNB)^1,2^. Compared to an axillary lymph node dissection (ALND), SLNB is associated with fewer late effects, but nevertheless has undesirable morbidity^3^. Thus, following more than a decade of omitting ALND if SLNB is negative, the search has been intensified for non-invasive prognosticators that might replace SLNB^4^. A predictive value of SLNB/ALN status has previously been explored for age at time of diagnosis, tumor size, multifocal tumor, lymphovascular invasion, tumor grade, and HER2-, progesterone-, and ER-receptor statuses^5–7^. These studies were generally aimed at developing algorithms that may predict ALN status and ultimately allow specific patient subgroups to completely avoid axillary surgery. The study by Reyal et al., which used surrogate markers (ER- and HER2-receptor statuses) for molecular subtype assignment of a large series of > 2500 early-stage breast cancer patients treated with conservative surgery including an SLNB, found both ER-positivity and the interaction covariate between ER- and HER2-statuses to be significant predictors of a positive sentinel node (SN)^8^. The study also developed a nomogram model for predicting ALN status; the algorithm was validated in an independent breast cancer cohort showing similar accuracy in predicting positive SLNB^9^. A recent study proposed a new algorithm to predict ALN status based on a selection of regression variables and confirmed age at time of diagnosis, tumor size, lymphovascular invasion, and molecular subtypes defined by surrogate markers as predictive factors^10^.

Intrinsic molecular subtypes have emerged as promising predictors of breast cancer recurrence; each subtype is a distinct biological entity and associated with specific prognostic and therapeutic features^11,12^. The pivotal studies proposed five original subclasses: Luminal A, luminal B, normal-like, HER2-enriched, and basal-like. Four of the subclasses can be distinguished by a 50‐gene molecular classifier (PAM50) which has also been developed as a commercial FDA approved platform^13^. A more recent taxonomy by Guedj et al. refined these molecular signatures by applying integrative transcriptomics analysis, leading to six stable molecular subtypes. Four of these, termed LumA, LumB, LumC, and NormL, are all ER-, progesterone (PR)-, and androgen-receptor (AR)-positive, while the fifth subtype, mApo, is AR-positive but ER- and PR-negative. The last subtype, BasL, is characterized by its lack of receptor expression (ER-, PR-, and AR-negative). The BasL subtype often comprises tumors that are so-called triple-negative, but these terms are not interchangeable^14^. In the six-subtype scheme, the *ERBB2*-amplified tumors are split between the mApo and the highly proliferative LumC subgroup^15,16^. Additionally, while the subtype names in the original five-subtype and the six-subtype schemes are similar, the underlying definition of each subtype is not identical (i.e. Luminal A of the five-subtype scheme is not identical to LumA of the six-subtype scheme).

To enable molecular insights, transcriptomics analysis has in recent years become a part of the standard of care for breast cancer patients in several clinical settings. In our regional hospital, all primary breast cancers are assigned with a molecular subtype from the scheme by Guedj et al. (named: CITBCMST) as part of our standard of care diagnostic pipeline^17^. This set-up provides a platform to test whether consecutive assignments of molecular subtypes may have additional predictive value for SLNB status.

One of the earliest studies assessing the predictive value of surrogate molecular subtypes showed that basal-like tumors were more likely to be ALN–negative^18^. Supportingly, a larger study based on > 4000 early stage breast cancers and surrogate molecular subtypes found the triple-negative subtype as a predictor of negative ALN status^19^. Conversely, triple-*positive* tumors have been shown to be predictive for a *positive* ALN status^20^. Later studies have confirmed the triple-negative subtype to be a predictor for negative ALN status^21,22^. However, a study by Lu et al. did not report any improvements in the predictive models for ALN status by applying the gene expression profiles from 129 tumor specimens^23^. Nonetheless, intrinsic subtypes should be recognized by expression signatures consisting of a minimum of 50 transcripts^13,24,25^. Thus, we hypothesized that transcriptomics-derived molecular subtypes based on consecutive primary breast cancer samples would be an optimized setting to predict SLNB status and subsequently permit specific patient subgroups to avoid axillary surgery.

## Results

### Distribution of SN status and subtypes

**Figure 1:**
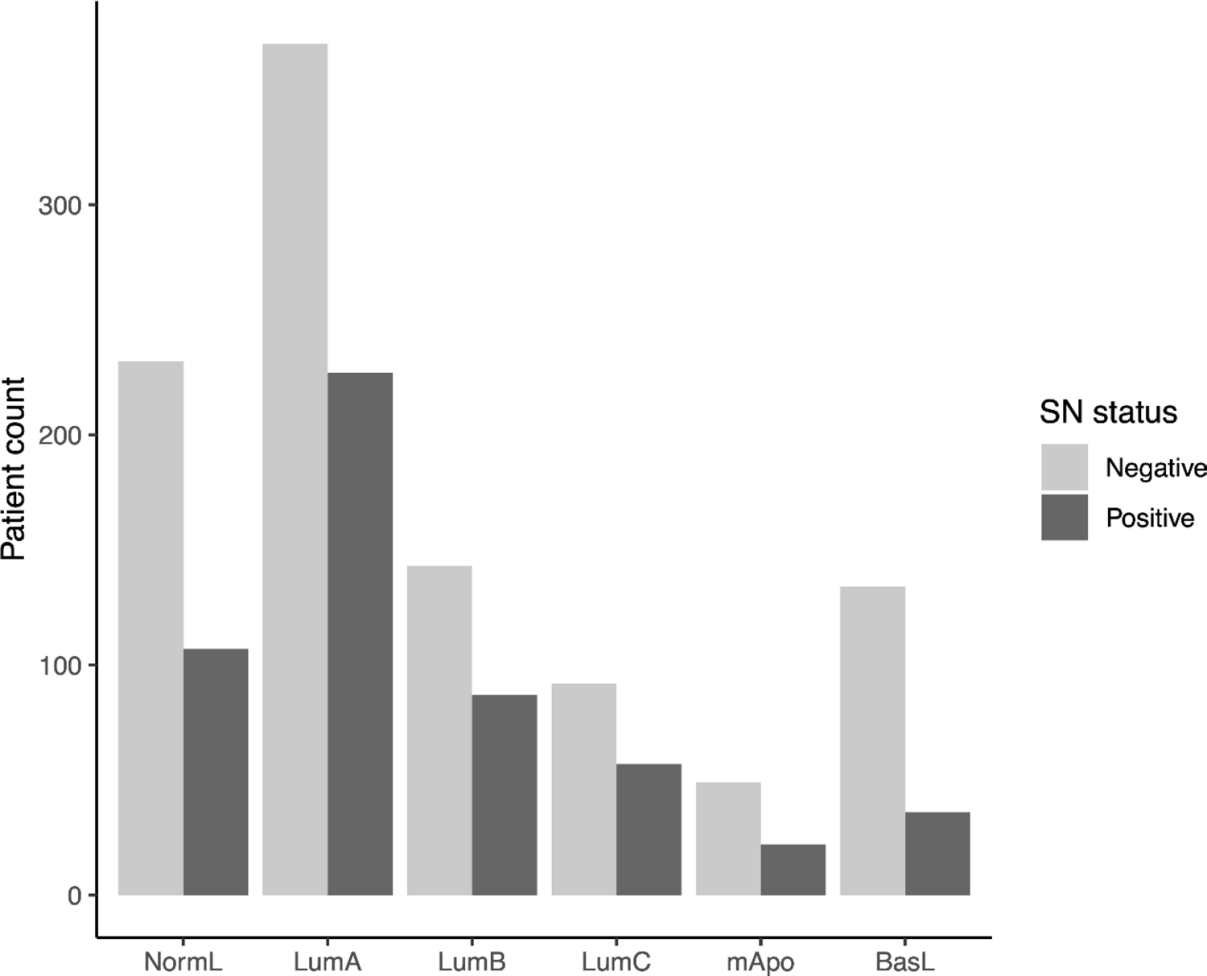
Distribution of molecular subtypes among the 1556 primary breast cancer patient samples > 10 mm and allocated for primary surgical procedure including a sentinel node (SN) examination. A total of 1020 patients had a SN-negative lymph node (pale grey) and 536 had a SN-positive lymph node (dark grey). The distribution of molecular subtypes (NormL, LumA, LumB, LumC, mApo, BasL) is illustrated at the x-axis and number of samples along the y-axis. Figure created using R^32^.

Of 3002 consecutive primary breast cancers (Nov. 2014-Sept. 2019), 1576 patients with a tumor size > 10 mm were allocated for primary surgical procedure including an SLNB and eligible for molecular subtyping. Following assessment of array results and tumor content based on the proliferative index, 20 samples were of insufficient quality for subtype allocation, leaving 1556 samples for further analysis. The overall molecular subtype distribution was NormL: 339; LumA: 597; LumB: 230; LumC: 149; mApo: 71 and BasL: 170, Table 1. The distribution of clinical and morphological characteristics among the six different molecular subtypes are listed in Table 1. Of the 1556 included patients, 1020 had a negative SN (NormL: 232; LumA: 370; LumB: 143; LumC: 92; mApo: 49; BasL: 134) and 536 had a positive SN (NormL: 107; LumA: 227; LumB: 87; LumC: 57; mApo: 22; BasL: 36), *p* = 0.001, Fig. 1.

**Table 1:** Distribution of molecular subtypes, menopausal status, tumor size, malignancy grade, and sentinel node (SN) status for 1556 patients.

Molecular subtype	NormL		LumA		LumB		LumC		mApo		BasL	
No. of patients (%)*	339 (22)		597 (38)		230 (15)		149 (10)		71 (5)		170 (11)	
**Menopause**												
Premenopause	87 (26)		117 (20)		69 (30)		42 (28)		12 (17)		68 (40)	
Postmenopause	252 (74)		480 (80)		161 (70)		107 (72)		59 (83)		102 (60)	
**Tumor size**												
11–20 mm	232 (68)		381 (64)		120 (52)		94 (63)		39 (55)		94 (55)	
> 20 mm	107 (32)		216 (36)		110 (48)		55 (37)		32 (45)		76 (45)	
**Grade**												
1	159 (47)		210 (35)		15 (7)		20 (13)		2 (3)		0 (0)	
2	168 (50)		323 (54)		113 (49)		71 (48)		16 (23)		14 (8)	
3	6 (2)		38 (6)		99 (43)		54 (36)		46 (65)		144 (85)	
N/A	6 (2)		26 (4)		3 (1)		4 (3)		7 (10)		12 (7)	
**SN status**												
SN-negative	232 (68)		370 (62)		143 (62)		92 (62)		49 (69)		134 (79)	
SN-positive	107 (32)		227 (38)		87 (38)		57 (38)		22 (31)		36 (21)	

	SN-	SN +	SN-	SN +	SN-	SN +	SN-	SN +	SN-	SN +	SN-	SN +
No systemic treatment	13 (6)	4 (4)	21 (6)	5 (2)	6 (4)	1 (1)	2 (2)	4 (7)	6 (12)	1 (5)	15 (11)	2 (6)
ET only	167 (72)	52 (49)	258 (70)	134 (59)	59 (41)	21 (24)	37 (40)	20 (35)	4 (8)	4 (18)	4 (3)	0 (0)
ET + CT	43 (19)	44 (41)	65 (18)	74 (33)	69 (48)	60 (69)	44 (48)	27 (47)	6 (12)	9 (41)	23 (17)	12 (33)
CT only	2 (1)	3 (3)	4 (1)	3 (1)	3 (2)	2 (2)	3 (3)	4 (7)	25 (51)	7 (32)	79 (59)	20 (56)
No registration (N/A)	7 (3)	4 (4)	22 (6)	11 (5)	6 (4)	3 (3)	6 (7)	2 (4)	8 (16)	1 (5)	13 (10)	2 (6)
(%) col												

*ET* endocrine therapy, *CT* chemotherapy.

**Figure 2:**
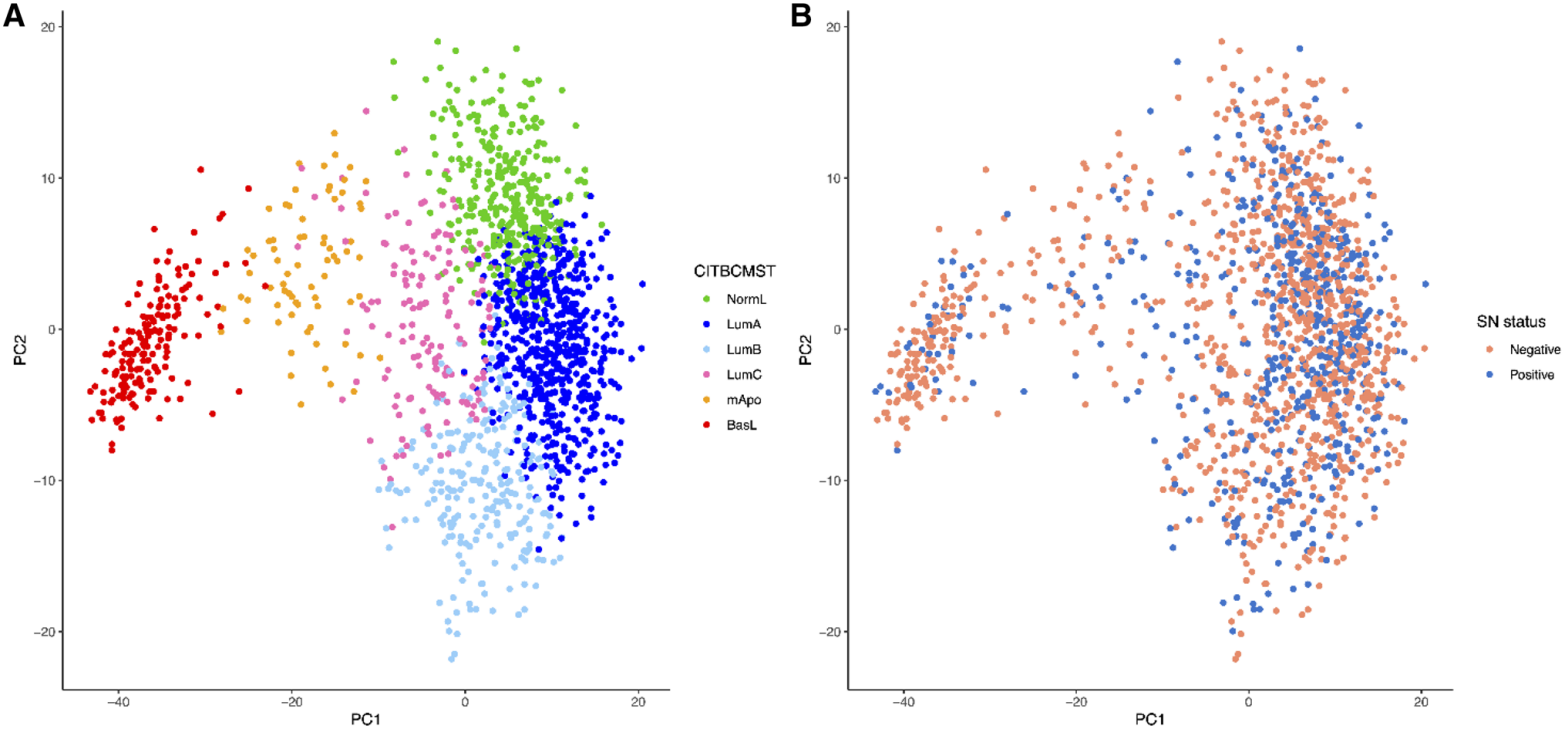
(**A**) depicts a principal component analysis (PCA) of the distribution of the six subtypes based on the 375 probe sets of the CITBCMST classifier; NormL (green), LumA (dark blue), LumB (light blue), LumC (pink), mApo (orange), BasL (red). (**B**) depicts the distribution of sentinel node status (**B**); orange color represents samples with a negative sentinel node; blue color represents samples with a positive sentinel node. Figure created using R^32^.

For a general overview of the 1556 samples, a principal component analysis (PCA) was generated to illustrate the assignments of subtypes (Fig. 2A) and the distribution of SN status (Fig. 2B). The figure clearly depicts the difference of the BasL subtype compared to the luminal subtypes.

When considering the two subtypes with *ERBB2*-amplified tumors (mApo and LumC) collectively, 36% of tumors were SN-positive. The same fraction of SN-positivity was observed in the combined set of *ERBB2*-normal luminal tumors (NormL, LumA and LumB). In contrast, merely 21% of the tumors with a BasL subtype had a positive SN status.

In terms of systemic treatment of the included patients, we observed that for the NormL and LumA subtypes, almost twice as many patients within the SN-positive fraction were treated with both endocrine- and chemotherapy (41 and 33%, respectively) relative to the SN-negative patients (19 and 18%, respectively), Table 1.

### Subtypes, clinical characteristics, and SN status

Considering the entire cohort, neither the patients’ menopausal status nor malignancy grade were associated to SN metastases (*p* = 0.11 and *p* = 0.12, respectively), whereas a clear association between tumor size and SN status (*p* < 0.0001) was found (Table 2). However, a tumor size of > 20 mm was only significantly associated to positive SN status among the samples assigned with luminal subtypes (NormL, LumA, LumB and LumC), and no significant association was identified for the samples assigned with mApo and BasL subtypes (data not shown). Since the BasL subtype was markedly different in the distribution of SN status compared to the other subtypes, heterogeneity between BasL vs. all other subtypes was investigated for association between SN status and menopausal status, malignancy grade and tumor size (Table 3). No statistically significant interaction was found, but we observed a trend (*p* = 0.054) for menopausal status, with a marked difference in the proportion of SN-positive patients among postmenopausal patients.

**Table 2:** Univariate logistic regressions comparing subtypes or clinicopathological features across subtypes.

	SN−	SN+	OR	95% CI	*p* value
**Subtype**					
NormL	232	107	*Ref*		0.001
LumA	370	227	1.33	[1.00;1.76]	
LumB	143	87	1.32	[0.93;1.87]	
LumC	92	57	1.34	[0.90;2.01]	
BasL	134	36	0.58	[0.38;0.90]	
mApo	49	22	0.97	[0.56;1.69]	
**Menopause**					
Premenopause	246	149	*Ref*		0.11
Postmenopause	774	387	0.83	[0.65;1.05]	
**Tumor size**					
11–20 mm	699	261	*Ref*		< 0.0001
> 20 mm	321	275	2.29	[1.85;2.84]	
**Grade**					
1	273	133	*Ref*		0.12
2	438	267	1.25	[0.56;1.69]	
3	260	127	1.00	[0.75;1.35]	
N/A	49	9	-	-	-
**Subtype (ERBB2)**					
NormL + LumA + B	745	421	*Ref*		0.96
mApo + LumC	141	79	1.01	[0.75;1.36]	

The odds ratio (OR) and 95% confidence interval (CI) for SN + vs. SN − is presented for each group compared to a reference (*Ref*). *p* values represent those of the χ^2^ test across all categories excluding unknowns.

**Table 3:** Logistic regressions for sentinel node status.

	BasL (*n* = 170)				Non-BasL (*n* = 1386)				
	SN−	SN+	OR [95% CI]	*P*_within_	SN−	SN+	OR [95% CI]	*P*_within_	*P*_interation_
**Menopause**									
Pre-menopause	47 (69)	21 (31)	*Ref*		199 (61)	128 (39)	*Ref*		0.054
Post-menopause	87 (85)	15 (15)	0.39 [0.18;0.82]	0.01*	687 (65)	372 (35)	0.84 [0.65;1.09]	0.19	
**Tumor size**									
11–20 mm	78 (83)	16 (17)	*Ref*		621 (72)	245 (28)	*Ref*		0.39
> 20 mm	56 (74)	20 (26)	1.74 [0.83;3.66]	0.14	265 (51)	255 (49)	2.44 [1.94;3.06]	< 0.0001*	
**Grade**									
1	0 (0)	0 (0)	-	-	273 (67)	133 (33)	*Ref*		0.85
2	11 (79)	3 (21)	*Ref*		427 (62)	264 (38)			
3	113 (78)	31 (22)	1.01 [0.26;3.83]	0.99	147 (60)	96 (40)	1.15 [0.87;1.53]	0.33	
N/A	10 (83)	2 (17)	-	-	39 (85)	7 (15)	-	-	
***BRCA1/2***									
Negative	68 (78)	19 (22)	*Ref*		N/A				
Positive	25 (86)	4 (14)	0.57 [0.18;1.85]	0.35					
N/A	41 (76)	13 (24)	-	-					
***BRCA1***									
Negative	75 (79)	20 (21)	*Ref*		N/A				
Positive	18 (86)	3 (14)	0.62 [0.17;2.34]	0.48					
N/A	41 (76)	13 (24)	-	-					
***BRCA2***									
Negative	86 (80)	22 (20)	*Ref*		N/A				
Positive	7 (88)	1 (13)	0.56 [0.07;4.78]	0.59					
N/A	41 (76)	13 (24)	-	-					

Multivariate regressions were used to analyze subtype (BasL or Non-BasL) together with each of menopausal state, tumor size, and tumor grade, respectively, and the odds ratio (OR) and 95% confidence interval (CI) within each subtype are reported. Wald tests were used to derive *p* values within each of the individual subtypes (*P*_within_) as well as for the interaction term (*P*_interaction_). Because only a limited number of non-BasL patients were screened for *BRCA1/2* carrier status, univariate logistic regression was performed for these features within the BasL subtype. Row-wise percentages in parentheses.

### Estrogen receptor activity and SN status

**Figure 3:**
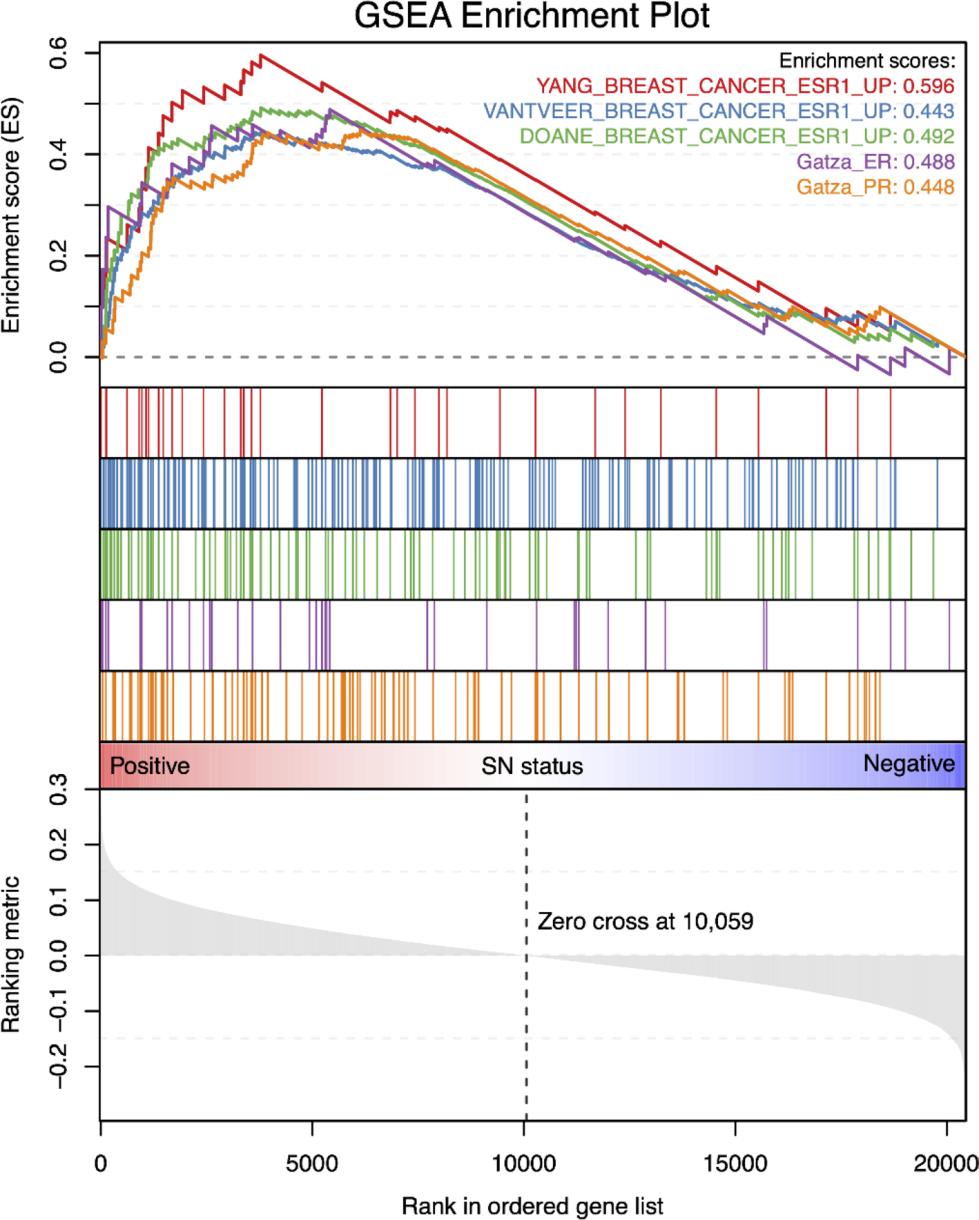
Barcode Enrichment Plot of the five signatures; Gatza_ER (purple), Gatza_PR (orange), “DOANE_BREAST_CANCER_ESR1_UP” (green), “VANTVEER_BREAST_CANCER_ESR1_UP” (blue) and “YANG_BREAST_CANCER_ESR1_UP” (red). All genes from the BasL samples (*n* = 170) are ranked along x-axis according to enrichment score (y-axis). There is a significant upregulation of the five investigated gene sets in patients with a positive SN status, and the ranked position of each gene within a signature is shown in the middle panel. Figure created using R^32^.

Subsequently, we sought to investigate if the level of ER-protein was associated with SN status in the BasL assigned patients and found that IHC-based ER status and SN status were not correlated, *p* = 0.36 (data not shown). Conversely, a comparison of the microarray-based *ESR1* expression vs. SN status did show a significantly higher *ESR1* level in the SN-positive patients (*p* = 0.01). This result lead us to conduct a GSEA using gene sets related to ER and PR expression which are upregulated in ER + vs. ER-human breast cancer samples (see Materials and Methods for gene signatures). We found a significant (false discovery rate-adjusted *p*-value < 0.05) upregulation for all five tested gene sets when comparing SN-positive BasL samples to SN-negative BasL samples (Fig. 3). Based on these results, we conclude that there is a correlation between hormone receptor activity and SN status within the BasL subtype.

### BRCA1/2-positive carriers and SN status

We sought to test if these findings were related to an enrichment of *BRCA1/2*-positive carrier status. Among the 170 patients assigned with a BasL subtype, 116 patients were screened for a *BRCA1/2*-predisposing variant. 29 were *BRCA* germline carriers (*BRCA1*-positive: 21, *BRCA2*-positive: 8). We found no significant association between *BRCA1/2* germline carriers and SN status (*p* = 0.35) (Table 3).

### Survival analysis

**Figure 4:**
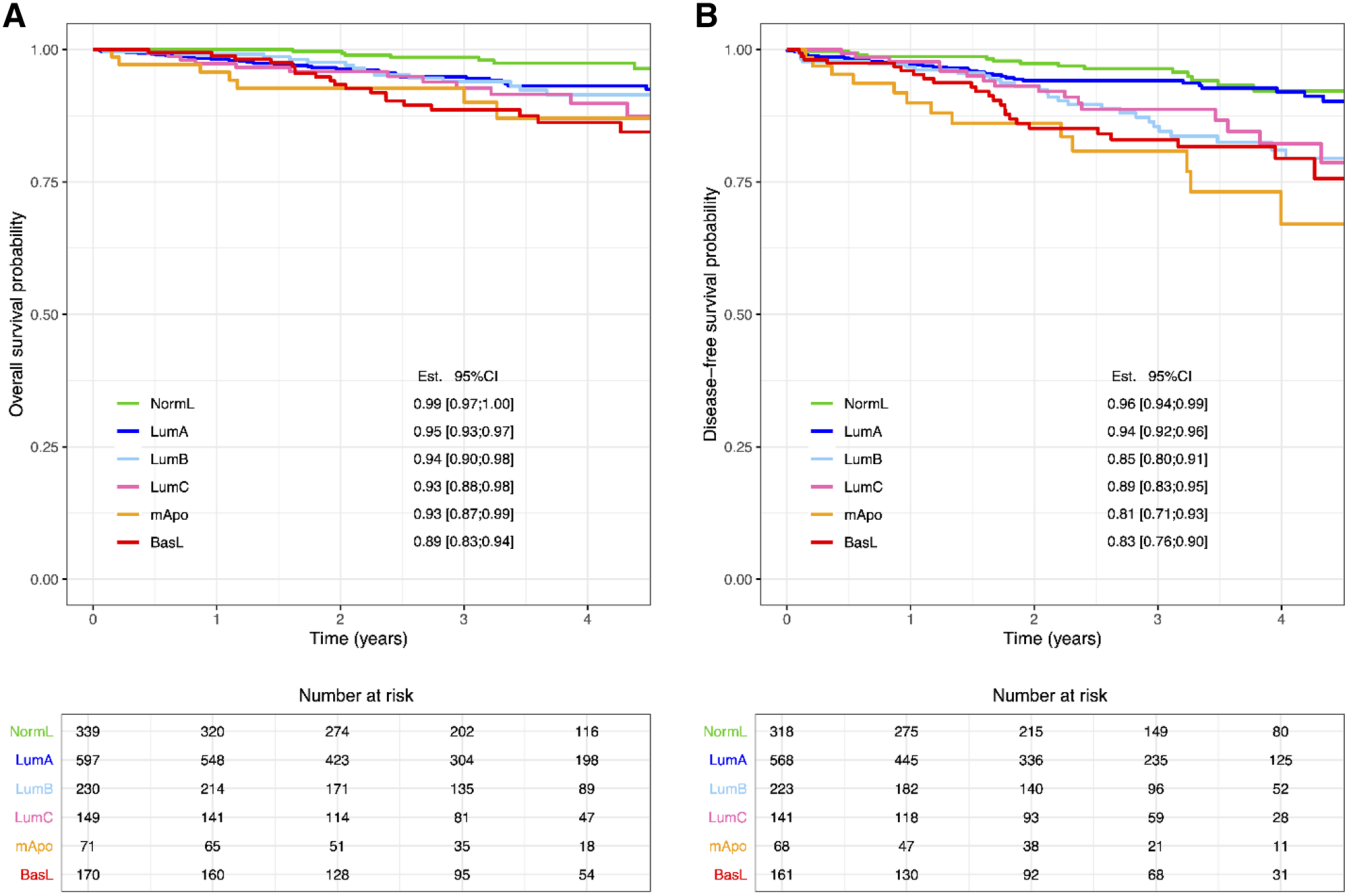
(**A**) shows the overall survival of the patient cohort according to the assigned molecular subtypes and (**B**) shows the disease-free survival. Molecular subtypes are color-coded; NormL (green), LumA (blue), LumB (light blue), LumC (pink), mApo (orange), BasL (red). The estimated survival probability (Est.) and the associated 95% confidence interval (95% CI) are shown at time = 3 years for each subtype. Figure created using R^32^.

Finally, we assessed the overall survival and disease-free survival of the patient cohort according to the assigned molecular subtypes and found a distributions of molecular subtypes in agreement with the overall consensus; LumA and NormL showed a favorable prognosis, both for OS and DFS, followed by LumB and LumC, and finally mApo and BasL showed the worst prognosis (Fig. 4, panels A and B).

**Figure 5:**
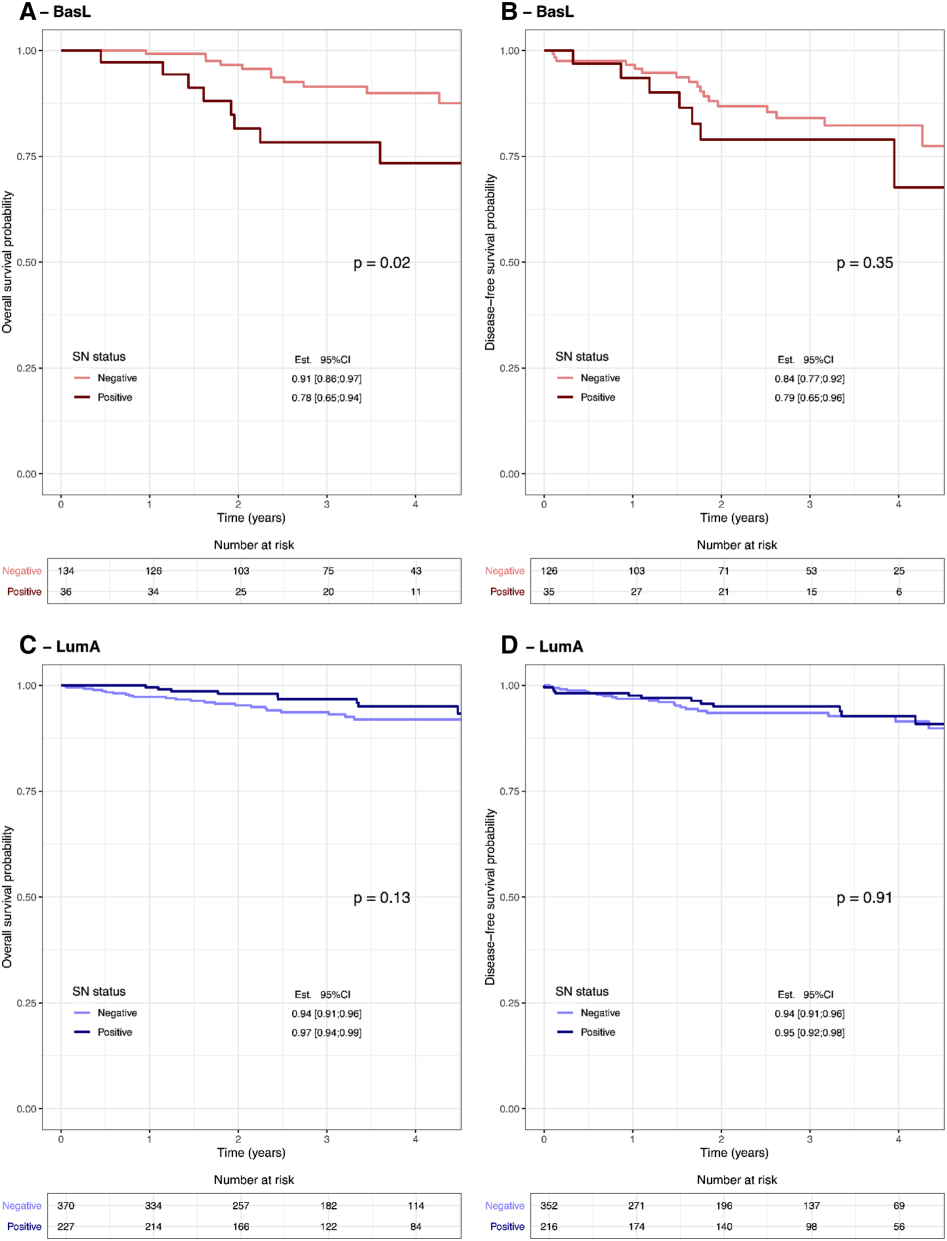
The overall survival of patients with a BasL subtype is depicted in (**A**). The disease-free survival of patients with a BasL subtype is depicted in (**B**). Sentinel node (SN) negative status (light pink), SN-positive status (dark red). The overall survival of patients with a LumA subtype is depicted in (**C**). The disease-free survival of patients with a LumA subtype is depicted in (**D**). SN-negative status (light blue), SN-positive status (dark blue). The estimated survival probability (Est.) and the associated 95% confidence interval (95% CI) are shown at time = 3 years for each SN status. Figure created using R^32^.

Subsequently, we examined the OS and DFS in the group of patients with BasL tumors according to SN status and found a significantly (*p* = 0.02) lower OS of patients with a positive SN vs. a negative SN (Fig. 5A, B). Evaluating the OS and DFS in the group of patients with LumA tumors according to SN status showed no significant difference between patients with a positive SN vs. a negative SN (Fig. 5C, D).

## Discussion

Our study confirmed a solid association between tumor size and SN status. However, this was only the case among the samples assigned with luminal subtypes (NormL, LumA, LumB and LumC). These findings are largely in agreement with the studies relying solely on surrogate markers^8,22^. In contrast, and to the best of our knowledge, it has not previously been shown that prediction of SN status is independent of tumor size in the BasL and mApo subtypes, when determined by multi-gene transcript-based signatures. Thus, applying a taxonomy that identifies a broader range of distinct subtypes may enable a more precise prediction of SN status.

The LumC and mApo subtypes are characterized by including the *ERBB2*-amplified tumors among other distinct molecular features like high proliferation (LumC) and overexpression of androgen-receptor (mApo), thus we speculated that an association to positive SN status was plausible. However, our results showed no evidence of subtypes including *ERBB2*-amplified tumors having a greater risk of a positive SN. This is not entirely in agreement with the previous study by Reyal et al. who found HER2-positive status in combination with ER-positive status, to be a significant predictor of a positive SN^8^. The discrepancies could be due to the differences in cohorts, since Reyal et al. included all patients with ALN metastasis, as well as the comparison of two different entities; single marker sample annotations as opposed to annotations by intrinsic subtypes derived from transcriptome-based signatures.

Interestingly, although it is well-established that BasL subtype has the poorest prognosis, BasL tumors in our cohort were not associated with SN-positive status. This agrees with reports where triple-negative receptor profiles have functioned as surrogate marker for BasL subtype. However, since this lack of association of BasL subtypes with positive SN status is rather peculiar, we looked deeper into the clinical behavior of patients with a BasL subtype and found a significant association if we segregated the BasL cohort into pre- and postmenopausal patients. The recent study from Bertucci et al.^26^ highlighted the differences among tumors assigned with a BasL subtype by comparing ER +/HER2- and ER-tumors and showed that ER +/HER2-BasL patients had a more similar clinical outcome to ER +/HER2-Luminal patients than to the ER-BasL. This is in line with the essence of our GSEA-results, which indicate that BasL-samples with an enrichment of estrogen-pathway signatures are overrepresented among the SN-positive patients. In other words, these samples are perhaps more like the luminal ER-positive subtypes in behavior, also regarding SN status. It has recently been shown that negative predictive factors for OS in *BRCA1/2* positive carriers included lymph nodes metastases^27^. We were not able to confirm these results since our cohort of *BRCA1/2* positive carriers merely comprises 29 patients, so statistical analysis is connected to great uncertainties. A limitation of our study is that the number *BRCA1/2* positive patients is to sparse to draw conclusions on possible association with SN status.

We found a clear association between OS and SN status in patients with a BasL tumor; SN-positive patients had a worse prognosis. Low risk of lymphatic spread in patients with triple-negative breast cancer has been shown before^2,4^. Several authors have suggested that axillary node status is losing its significance as a prognostic marker with the increasing use of molecular subtypes, especially in patients with triple-negative breast cancer, and axillary staging might not be mandatory^28^. Irrespective of node status the vast majority of patients with a BasL breast cancer will be recommended (neo)adjuvant chemotherapy and may not obtain a beneficial impact from postmastectomy radiotherapy if node positive^29^. However, the results of SUPREMO must be awaited before this can be further clarified^30^. Despite the low risk of lymphatic spread in patients with BasL subtypes, lymph node metastases in these patients may still, according to our results, be of prognostic significance.

The major limitation of our study is the time of follow-up which is only four years. Particularly in patients with ER-positive breast cancer, recurrence can occur up to twenty years after time of primary diagnosis^31^. Thus, evaluating OS and DFS for patients with ER-positive cancers in our study has limited significance. Hence, at this early stage of the follow-up period, we found no prognostic association of patients with a LumA subtype and a positive SN biopsy, neither in OS- nor DFS-analysis.

In the present study we sought to test whether consecutive assignments of molecular subtypes may have additional prognostic value as predictive markers for positive SN. The design of our study is unique as it relies solely on transcriptomics-based molecular subtypes generated from consecutive breast cancer biopsy samples collected and analyzed in a prospective real-life clinical setting during a period of five years. Overall, the results derived from the present study are based on a reasonable number of patients (> 1500) and can be translated into any breast cancer clinic.

Although the molecular profiles of breast cancers are heterogenous, recent decades of investigations have proven how well multi-gene profiles cluster into separate intrinsic molecular subtypes. Each individual subtype has a distinct molecular profile that provides insights to a targeted treatment strategy and each subtype is associated with clinical characteristics and specific prognosis. However, axillary status and staging of the patient is a major prognostic factor in breast cancer management and the application of SN status guides the level of adjuvant treatment and radiotherapy for the bigger part of primary breast cancer patients.

In conclusion, we have shown that molecular subtypes are associated with SN status and a BasL subtype is a significant predictor of a SN-negative status. Hence, when assessing SN status, we can conclude that the BasL subtype is indeed a different entity compared to luminal subtypes. However, we have also shown that BasL samples with an enrichment of estrogen-pathway signatures are overrepresented among the SN-positive patients and thus show multiple overlapping clinical features with the luminal ER-positive subtypes.

## Materials and methods

### Patients and tumor samples

The cohort includes 3002 consecutive registrations from female primary breast cancer patients, clinically and diagnostically assessed at Rigshospitalet, Copenhagen University Hospital during the period from November 2014 until September 2019. Tumor specimens were subjected to molecular subtyping as a part of routine diagnostic work-up. This register-based study was conducted with approval of the Danish Data Protection Agency (jr. no.: 2012-58-0004) and Danish Breast Cancer Group (jr. no.: DBCG-2015-14). The study did not include any contact with patients nor use of biological material, and thus ethical approval, including the need to obtain informed consent, was explicitly waived by the Ethical Committee of the Capital Region of Denmark. None of the authors could access identifying patient information when analyzing the data. Fresh tumor specimens, extracted during surgery, were inspected by pathologists, and tumor biopsies of around 100 mg were stored in RNALater (Thermo Fisher Scientific, Waltham, MA, USA). RNA was isolated using the AllPrep DNA/RNA purification kit (Qiagen, Hilden, Germany). The integrity of the RNA was measured using the Agilent RNA 6000 Nano Kit on an Agilent 2100 Bioanalyzer (Agilent Technologies, Inc., Santa Clara, CA, USA). RNA was reverse transcribed and used for cRNA synthesis, labeling and hybridization with GeneChipVR Human Genome U133 Plus 2.0 Array (Affymetrix, Santa Clara, CA, USA) according to the manufacturer’s protocol. In short, arrays were washed and stained with phycoerytrin conjugated streptavidin using the Affymetrix Fluidics Station 450 and scanned in the Affymetrix GeneArray 3000 7G scanner to generate fluorescent images. Probe intensity files (.CEL files) were generated in the GeneChip Command Console Software (AGCC; Affymetrix, Santa Clara, CA, USA).

### Sentinel node status

Preoperative axillary sonography was performed in all patients to identify lymph node metastases. In case of suspicious lymph nodes by sonography, fine needle aspiration cytology (FNAC) was performed. If malignant tumor cells were found by FNAC, an immediate ALN dissection was offered, and these patients were excluded. In the remaining patients SLNB was performed. Prior to surgery, 99mTc labeled NanoColl was injected subareolarly, and the blue dye, Patent Blue, was injected at the tumor site or the subareolar region. All radioactive and/or stained lymph nodes as well as lymph nodes suspicious by palpation were removed as sentinel nodes for histopathological examination, including multisectioning and immunohistochemical (IHC) staining. Metastases were staged according to 8th edition of the American Joint Committee on Cancer staging manual where macrometastases are defined as metastases > 2 mm, micrometastases as metastases > 0.2 mm to ≤ 2.00 mm, while isolated tumor cells (ITC) are defined as deposits of cells ≤ 0.2 mm or ≤ 200 cells. Patients were considered SN-positive if macro- or micrometastases were found in the SN, and SN-negative if isolated tumor cells or no metastases were found, but only patients with macrometastases were offered ALND.

### Microarray analysis

The probe level data (.CEL files) were transformed into expression measures using R version 3.2.5^32^. For each sample, the raw intensity .CEL file was preprocessed together with 30 existing breast cancer samples from Rigshospitalet by quantile normalization, and probe summaries were extracted via robust multi-array average (RMA) using the affy package^33^. Subsequently, ComBat^34^ from the sva package^35^ was applied for batch correction of 12 of the reference samples and the sample of interest together with the CITBCMST^16^ core set (*n* = 355), as presented in Rossing et al.^17^. Sample origin was used as batch and initially predicted CITBCMST subtypes (determined using the citbcmst R-package) acted as covariates^16^.

For each sample, a subtype was subsequently assigned using the CITBCMST tool^16^, which assigns samples to one of six subtypes—BasL, mApo, LumA, LumB, LumC, or NormL—using a distance-to-centroid approach relying on expression of 375 probe sets. In the standard CITBCMST tool, a confidence score for each subtype is also provided. If a sample is close to a single centroid, it is labeled as “core”, whereas if it is close to multiple or no centroids, it will be labeled as “mixed” or “outlier”, respectively. In this study, all outlier samples (*n* = 20) were removed from further analysis since they classified as normal tissue due to contamination of normal breast tissue cells^36^. All mixed samples were labeled based on the single closest centroid, leaving only six subtypes in the downstream data analysis. Visualizations were generated using the ggplot2 (https://ggplot2.tidyverse.org) and cowplot (https://CRAN.R-project.org/package=cowplot) packages.

### Estrogen receptor protein immunohistopathological analysis

Analyses for ER were performed by immunohistochemistry (IHC) using tissue micro array technique (TMA), with two cores of 2 mm from the invasive front of each tumor, as previously reported^37^. Staining for ER (SP1, diluted 1:25) from Ventana Medical Systems was carried out according to the manufacturer’s instructions. Scoring of ER protein was semi-quantitative with a positive cutoff point of ≥ 1% for ER-positive tumors.

### Blood sample and germline mutation screening

Genomic DNA was isolated using the ReliaPrep Large Volume HT gDNA Isolation Kit (Promega, Madison, WI, USA) and a Tecan Freedom EVO HSM2.0 Workstation according to the manufacturer’s instructions. Mutation screening was done using the breast cancer-predisposing gene-panel as previously described^38^. Sequencing was performed on a MiSeq (Illumina, San Diego, CA, USA) to an average depth of at least 100. Sequencing data were analyzed using Sequence Pilot (JSI Medical Systems, Ettenheim, Germany), where variants are called if the non-reference base frequency was above 25%. Variants are numbered according to the following GenBank accession numbers: NM_007294 (*BRCA1*) and NM_000059 (*BRCA2*) using the guidelines from the Human Genome Variation Society (www.hgvs.org/mutnomen). All class 3–5 variants were verified by Sanger sequencing on an ABI 3730 DNA Analyzer using DNA purified from a second blood sample.

### Overall and disease-free survival

The cut-off date for the survival parameters was May 31, 2020. Overall survival (OS) was defined as the interval from the date of primary surgical procedure until death, irrespective of cause. Complete follow-up until cut-off date was achieved by linkage to the Danish Central Population Registry, with nine patients being censored due to emigration. A total of 100 deaths were registered. Estimated median potential follow-up was 3.37 years (IQR [2.12;4.54]). Disease-free survival (DFS) was defined as the interval from primary surgical procedure until a first event, including recurrence (60 patients), contralateral breast cancer (seven patients), another malignancy (22 patients), and death as first event (44 patients). Seventy-seven patients were not enrolled in a protocol and consequently had no follow-up. These were excluded from the DFS-analysis.

### Statistical analysis

Information on age at diagnosis, menopausal status, tumor size, grade of malignancy, ER-status, SN status, adjuvant treatment, clinical follow-up, and vital status was obtained from the clinical database of DBCG. *BRCA1* and *BRCA2* class 4–5 variants were considered as “positive” status, and patients with at least one positive status were labeled “*BRCA1/2* positive”. Associations between SN status and other characteristics were analyzed by χ^2^ tests. Univariate logistic regression analysis was applied to assess odds ratios and corresponding 95% confidence intervals for SN-positive status over SN-negative and using the Wald test. For variables with an unknown category, patients with unknown values were excluded from the tests. Multivariate logistic regression analysis was applied to assess heterogeneity according to BasL vs. Non-BasL for each of menopausal status, tumor size, and grade, respectively. Each interaction was tested in a separate model, including subtype and the parameter of interest. Follow-up time was quantified in terms of a Kaplan–Meier estimate of potential follow-up. OS and DFS was analyzed unadjusted by the Kaplan–Meier method, and groups were compared using the log-rank test. All *p* values are two-tailed, and level of significance set to 0.05. Statistical analyses were performed using the R software^32^.

### BasL-specific analysis

All .CEL files from BasL patients (*n* = 170) were loaded into R 3.6.1^32^ using justRMA from affy^33^. For processing Entrez gene-level expression, the BrainArray^39^ Custom CDF v. 24 was used, and otherwise default parameter settings were used. Entrez Gene IDs were subsequently translated to HGNC symbols using the BrainArray annotation from hgu133plus2hsentrezg.db. After filtering out data with missing symbols, expression data for 20,418 genes remained. Differential expression analysis was carried out for all genes using limma^40^, and a label-permuting Gene Set Enrichment Analysis (GSEA) was carried out using fgseaLabel from the fgsea package^41^ with 100,000 permutations and default settings. The labels for both tests were the per-sample SN status. For this, we used the ER- and PR-sets from Gatza et al.^42^ as well as the following three MSigDB^43^ gene sets: “DOANE_BREAST_CANCER_ESR1_UP”^44^, “VANTVEER_BREAST_CANCER_ESR1_UP”^45^, and “YANG_BREAST_CANCER_ESR1_UP”^46^. Barcode enrichment plots for the GSEA analysis was generated using a modified version of the script: https://github.com/PeeperLab/Rtoolbox/blob/master/R/ReplotGSEA.R. The modified version is available on GitHub: https://github.com/cbligaard/Rtools. Tests for simple comparisons regarding receptor status or expression level and SN status and menopausal status were carried out using Pearson’s Chi-squared and two-tailed Wilcoxon rank-sum tests in R^32^.

### Data availability

Expression profiles are available in the online data repository Gene Expression Omnibus (GEO).

### Method statement

All methods were carried out in accordance with relevant guidelines and regulations.
